# Draft genome assembly and annotation of *Coelastrella* sp. UAM-C/S07 from Chalchoapan Lake, Mexico

**DOI:** 10.1128/mra.01441-25

**Published:** 2026-04-23

**Authors:** Mauricio Carrasco-González, Adrian A. Estrada-Graf, Diego A. Esquivel-Hernández, Laura González-Resendiz, Marcia Morales-Ibarría

**Affiliations:** 1 Posgrado en Ciencias Naturales e Ingeniería, Universidad Autónoma Metropolitana-Cuajimalpa, Mexico City, Mexico; 2 Marine Laboratory, Duke University, Beaufort, North Carolina, USA; 3 Departamento de Procesos y Tecnología, Universidad Autónoma Metropolitana-Cuajimalpa 103801 , Mexico City, Mexico; University of California Riverside 8790, Riverside, California, USA

**Keywords:** chlorophyte genomics, microalgae, *Coelastrella*, carotenoids, biofuels, long-read sequencing

## Abstract

In this study, the genome of the microalgal strain *Coelastrella* sp. UAM-C/S07 was analyzed to assess its potential as a strain capable of producing high-value carotenoids and fatty acids. A total of 9,319 coding sequences (CDSs) were identified, distributed across 1,065 contigs assembled using a *de novo* approach.

## ANNOUNCEMENT

Mitigating atmospheric CO₂ is a major environmental challenge, and microalgae act as efficient carbon sinks that often surpass terrestrial plants in carbon fixation (1). Microalgal biomass also provides a sustainable source of renewable energy and high-value metabolites (2, 3). The genus *Coelastrella* is promising due to its capacity to produce carotenoids, such as astaxanthin and canthaxanthin, and to accumulate lipids suitable for biodiesel (4–6). However, genomic resources for this genus remain limited.

A *Coelastrella* sp. strain previously isolated from Chalchoapan Lake, Veracruz, Mexico (18.41,611° N, 95.13,417° W) (7), was used for genomic analysis. The strain was established as a monoclonal culture from a single isolated cell and maintained in BG11 medium for 72 h at 35°C, 100 rpm, and 100 μmol photons m⁻² s⁻¹ prior to DNA extraction (8).

Genomic DNA was extracted using the DNeasy PowerSoil Kit (QIAGEN, Germany) following the manufacturer’s protocol with minor modifications. DNA quality was assessed with a Thermo Fisher Nanodrop One (OD260/280 and OD260/230), and integrity was verified by agarose gel electrophoresis (9).

Whole-genome sequencing was performed on a MinION R10.4.1 flow cell (mK1b device, FLO-MIN114) with the Rapid Barcoding Kit V14 (SQK-RBK114.24; Oxford Nanopore Technologies, Oxford, UK), generating 1,031,325 reads (200–173,656 bp). Read quality was evaluated with FastQC (v0.74) (10), and Filtlong (v0.3.1) (11) was used for Q20 filtering, yielding 598,477 reads with an N50 of 3,751 bp. Assembly was generated with Flye (v2.9.6) (12), polished with three rounds of Racon (v1.4.3) (13) and one round of Medaka (14), followed by removal of redundant contigs using purge_dups (v1.2.6) (15). Contaminant and chimeric sequences were removed using BlobToolkit (v4.4.6) (16), Kraken2 (Standard Database) (v2.17.0) (17), and FCS-GX (v0.5.5) (18). Assembly quality and completeness were assessed with QUAST (v5.2.0) (19) and BUSCO (v5.5.0) (20).

The final assembly comprised 1,065 contigs totaling 89.9 Mb, with an N50 of 218 kb, L50 of 127, GC content of 52.05%, and BUSCO completeness of 91%. Structural gene prediction was performed using GeMoMa (v1.9) (21), with genome annotations from *Scenedesmus glucoliberatum* PABB004 (22) and *Scenedesmus* sp. NREL 46B-D3 (23) as reference species for predicting protein-coding genes.

Functional annotation was performed using InterProScan (v5.76-107.0) (24), eggNOG-mapper (v2.1.13) (25), and BLASTp (v2.1.16) (26) searches against the UniProtKB/Swiss-Prot database. Detailed annotation coverage and completeness metrics are summarized in Table 1.

**TABLE 1:** Assembly and annotation summary for *Coelastrella* sp. UAM-C/S07

Category	Metric	Value
Genome assembly	Assembly size	89.9 Mb
	Number of contigs	1,065
	N50	218 kb
	L50	127
	GC content	52.05%
Genome annotation	BUSCO completeness	Assembly: 91%; annotation: 87%
	Predicted protein-coding genes	9,319
	Annotation coverage	InterProScan: 8,645 (92.8%) COG: 7,139 (76.6%) Swiss-Prot: 5,311 (57.0%) KEGG: 4,649 (49.9%) GO terms: 1,302 (14.0%)
	Total ncRNA loci	119 (54 tRNAs, 7 rRNAs, 3 snRNAs, 21 miRNA-family elements, 33 isrG-like small RNAs)

Non-coding RNA annotation was performed with Infernal (v1.1.5) (27), and full-length rRNA genes were independently predicted with Barrnap (v0.9) (28). The annotation identified 11 genes involved in the carotenoid biosynthesis pathway, including beta-carotene 3-hydroxylase (EC 1.14.15.24) and 93 genes associated with lipid metabolism, such as malonyl-CoA S-malonyltransferase (EC 2.3.1.39) and delta-12 desaturase (EC 1.14.19.6). A summary of genome assembly and annotation metrics, including functional annotation coverage and completeness statistics, is provided in Table 1.

This draft genome assembly represents a valuable resource for future research on microalgae as producers of high-value compounds, such as pigments and lipids, and for advancing studies in genome biology and metabolic engineering.

## Data Availability

This Whole Genome Shotgun project has been deposited at DDBJ/ENA/GenBank under the accession JBSVDD000000000. The version described in this paper is version JBSVDD010000000. Raw sequencing data have been deposited in the NCBI Sequence Read Archive (SRA) under the accession SRR36252716, BioProject accession PRJNA1369635, and Biosample SAMN53397293. The corresponding genome annotation (gene models, CDSs, and predicted proteins), including the 18S rRNA gene sequence used for molecular identification of the strain, has been uploaded to NCBI and is also available via Zenodo (https://doi.org/10.5281/zenodo.18555187).
